# Acute Kidney Injury Associated with Anticancer Therapies: Small Molecules and Targeted Therapies

**DOI:** 10.34067/KID.0000000566

**Published:** 2024-08-26

**Authors:** Jaya Kala, Teresa Joseph, Marta Pirovano, Roberta Fenoglio, Laura Cosmai

**Affiliations:** 1 Division of Nephrology, Department of Internal Medicine, University of Texas Health Science Center-McGovern Medical School, Houston, Texas; 2 Section of Nephrology, Department of Internal Medicine, Yale School of Medicine, New Haven, CT; 3 Department of Biomedical and Clinical Sciences, University of Milan, Milan, Italy; 4 Dana-Farber Cancer Institute, Harvard Medical School, Boston, Massachusetts; 5 University Center of Excellence on Nephrological, Rheumatological and Rare Diseases (ERK-net, ERN-Reconnect and RITA-ERN Member) including Nephrology and Dialysis Unit and Center of Immuno-Rheumatology and Rare Diseases (CMID), Turin, Italy; 6 Coordinating Center of the Interregional Network for Rare Diseases of Piedmont and Aosta Valley (North-West Italy), San Giovanni Bosco Hub Hospital, ASL Cittàdi Torino, Turin, Italy; 7 Department of Clinical and Biological Sciences of the University of Turin, Turin, Italy; 8 Onconephrology Outpatient Clinic, Nephrology and Dialysis Unit, ASST Fatebenefratelli Sacco, Milan, Italy

**Keywords:** AKI, albuminuria, BP, chemotherapy, drug nephrotoxicity, hypertension, kidney biopsy, nephrotic syndrome, nitric oxide, proteinuria

## Abstract

Molecular targeted therapy has revolutionized cancer treatment by significantly improving patient survival compared with standard conventional chemotherapies. The use of these drugs targets specific molecules or targets, which block growth and spread of cancer cells. Many of these therapies have been approved for use with remarkable success in breast, blood, colorectal, lung, and ovarian cancers. The advantage over conventional chemotherapy is its ability to deliver drugs effectively with high specificity while being less toxic. Although known as “targeted,” many of these agents lack specificity and selectivity, and they tend to inhibit multiple targets, including those in the kidneys. The side effects usually arise because of dysregulation of targets of the inhibited molecule in normal tissue. The off-target effects are caused by drug binding to unintended targets. The on-target effects are associated with inhibition toward the pathway reflecting inappropriate inhibition or activation of the intended drug target. Early detection and correct management of kidney toxicities is crucial to preserve kidney functions. The knowledge of these toxicities helps guide optimal and continued utilization of these potent therapies. This review summarizes the different types of molecular targeted therapies used in the treatment of cancer and the incidence, severity, and pattern of nephrotoxicity caused by them, with their plausible mechanism and proposed treatment recommendations.

## Introduction

**Figure 1:**
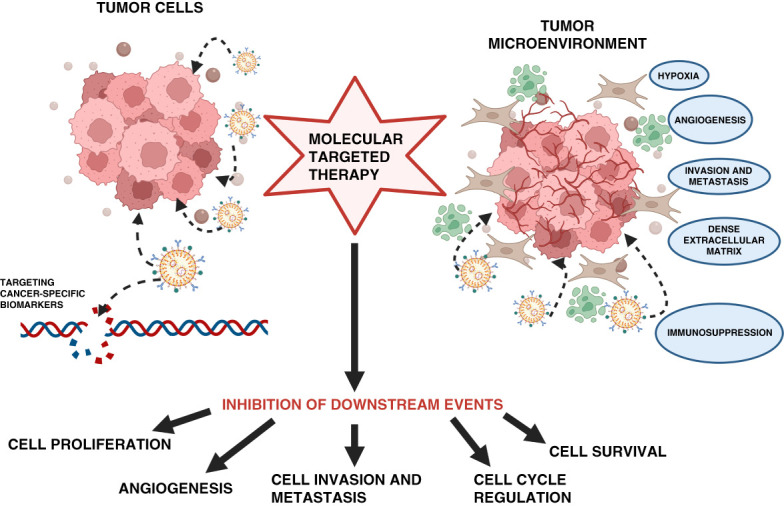
**Overview of the molecular targeted therapy mechanism.** Molecular targeted therapy on cancer focuses on targeting specific cancer-associated molecules that are highly expressed in cancer cells or by modulating the tumor microenvironment related to tumor vasculature, metastasis, or hypoxia. These lead to the inhibition of tumor cell proliferation, cell survival, cell cycle progression, angiogenesis, and cell invasion and metastasis. (Adapted from ref. 1). (Figure generated using BioRender.)

**Figure 2:**
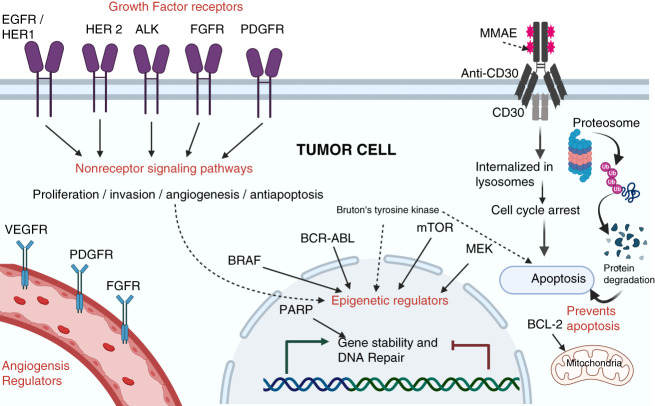
**Molecular targets in tumor cells that act as sites of action for chemotherapy.** Adapted with permission from ref. 2. ALK, anaplastic lymphoma kinase; BCL-2, B-cell lymphoma 2 gene; BCR-ABL, gene sequence; BRAF, v-Rapidly accelerated fibrosarcoma viral oncogene homolog B1; CD30, lymphocyte activation antigen; EGFR, epidermal growth factor receptor; FGFR, fibroblast growth factor receptors; HER, human epidermal growth factor; MEK, mitogen-activated protein kinase; MMAE, monomethyl auristatin E; mTOR, mechanistic target of rapamycin; PARP, poly-adenosine diphosphate-ribose polymerase; PDGFR, platelet-derived growth factor receptor; VEGFR, vascular endothelial growth factor receptor. (Figure generated using BioRender.)

Molecular targeted therapies have greatly improved survival for many types of malignancies. Unlike conventional chemotherapy, targeted therapies block the growth and spread of cancer by interfering with specific molecules involved in tumor growth and progression (Figure 1). The agents are classified into small molecules, monoclonal antibodies (mAbs**)**, immunotherapeutic cancer vaccines, and gene therapy (Table 1).^1^ They target growth factors, cell surface antigens, receptors/signal transduction pathways, cell cycle proteins, and modulators of apoptosis and angiogenesis, among many others (Figure 2).

**Table 1:** Types of molecular targeted therapies^1^

Types of Molecules	Mechanism/Site of Action	Examples
Small molecules	TKIs	Sorafenib, crizotinib
	Proteasome inhibitors	Carfilzomib, bortezomib, ixazomib
	CDK inhibitors	Ribociclib, palbociclib
	PARP inhibitors	Rucaparib, olaparib, niraparib
mAbs	Target extracellular proteins interrupting interactions between the receptor and ligand, thereby inhibiting tumor growth	Trastuzumab, brentuximab, adotrastuzumab-emtansine, cetuximab, bevacizumab, nivolumab, pembrolizumab, daratumumab
Therapeutic cancer vaccines	Target immune-mediated antitumor response	Proteins targeted by vaccines include oncoproteins, oncofetal antigens, viral proteins, hepatitis C virus
Gene therapy	Introduce genetic material DNA or RNA into cancer cells to destroy or inhibit their growth	Immunotherapy, oncolytic virotherapy, targeted genomic intervention, gene editing and gene transfer

CDK, cyclin-dependent kinase; mAb, monoclonal antibody; PARP, poly-adenosine diphosphate ribose polymerase; TKI, tyrosine kinase inhibitor.

**Figure 3:**
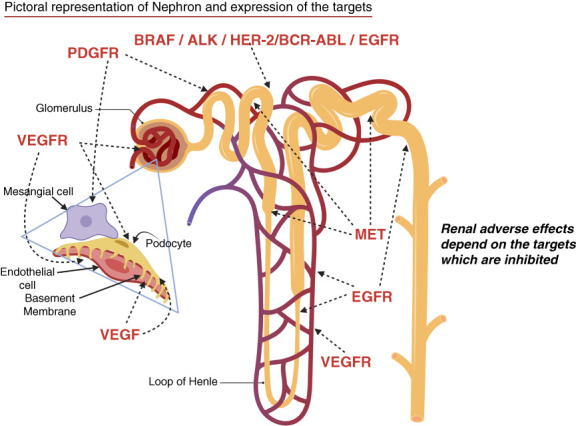
**Molecular targets expressed in the kidney**. Adapted with permission from ref. 2. MET, mesenchymal–epithelial transition; VEGF, vascular endothelial growth factor. (Figure generated using BioRender.)

They, however, lack specificity and selectivity while inhibiting multiple targets. Many of the targeted pathways are expressed in kidneys. Data from phase 2 and 3 clinical trials have revealed specific toxicities involving the skin, cardiovascular system, and kidneys.^3^ This is mainly due to coexpression of targets on membranes and intracellular pathways on both the tumor cells and normal cells (Figures 2 and 3).^3^ Multitarget cancer therapies that simultaneously target multiple signaling pathways/receptors, while more efficacious, are associated with a higher incidence of off-target toxicities.^3^

The toxicities from targeted therapies range from damage to glomeruli, tubules, interstitium, or microvasculature manifesting as asymptomatic proteinuria to kidney failure. Review of Food and Drug Administration Adverse Event Reporting System (FAERS) data (2011–2015) showed a high number of kidney adverse events with novel targeted therapies.^4^ Metabolic disturbances were found in 42.2%, kidney impairment in 42.2%, and hypertension (HTN) in 10.5% of patients.

The risk of direct kidney injury of these agents is higher with concomitant use of nephrotoxic agents, intrinsic kidney disease, volume depletion, or obstructive lesions. In this review, we attempt to highlight kidney injury of some targeted chemotherapies, their mechanism of action, and proposed treatment options. Immunotherapy has not been included in this review.

## Vascular Endothelial Growth Factor/Vascular Endothelial Growth Factor Receptor Inhibitors

### Mechanism of Action/Current Indications

Antiangiogenic therapy targeting vascular endothelial growth factor and its receptors (VEGFR) has revolutionized the treatment of various malignancies (renal cell carcinoma [RCC], endometrial cancer, thyroid cancer, colorectal cancer).

These drugs inhibit the VEGF pathway, which play a crucial role in tumor angiogenesis, fundamental for tumor growth and metastasis.^5^

**Figure 4:**
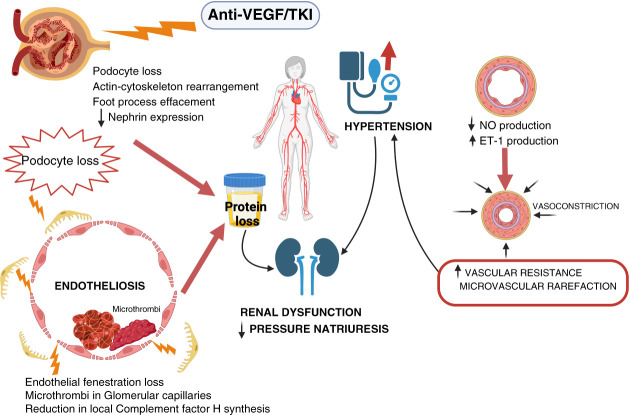
**Potential mechanisms of kidney injury and HTN induced by antiangiogenic treatment.** Systemic vascular endothelial cell dysfunction and localized kidney effects (glomerular endothelial cells and podocytes) of antiangiogenesis drugs lead to clinical kidney syndromes. NO, nitric oxide; ET, endothelin; HTN, hypertension; TKI, tyrosine kinase inhibitor. (Figure generated using BioRender.)

Several strategies are available for inhibiting VEGF/VEGFR signaling. mAbs, such as bevacizumab, or fusion proteins, such as aflibercept, can inhibit VEGF, while fully humanized Abs, such as ramucirumab, target VEGFR, and multi-tyrosine kinase inhibitors (mTKIs), such as sunitinib, cabozantinib, lenvatinib, and axitinib, block VEGFR's intracellular tyrosine kinase activity. mTKIs are not only directed against the VEGF/VEGFR pathway but also inhibit various cellular processes involved in tumor growth and angiogenesis (*e.g*., platelet-derived growth factor receptor, fibroblast growth factor receptor [FGFR], epidermal growth factor receptor [EGFR], and c-mesenchymal–epithelial transition [cMET]).^5^ The toxicity of targeted drugs depends on the specific target of the drug itself, and for this reason, adverse events are referred to as class effects. Inhibition of VEGF signaling causes disruption of the delicate balance of kidney microvasculature, leading to alterations in kidney endothelium and subsequent proteinuria, HTN, AKI, and CKD (Figure 4).^6^

### Kidney Toxicity

HTN is a frequent adverse event with an incidence ranging from 30% to 80%, depending on the specific drug, dosage, and comorbidities. Risk factors include preexisting HTN, age, and obesity. Mechanisms by which antiangiogenic agents induce HTN include microcapillary rarefaction; suppression of endothelial nitric oxide synthase; sodium retention; and increased synthesis of Endothelin 1, a potent vasoconstrictor agent.^7^

The most common nephrological adverse event is proteinuria. Incidence and severity vary among studies because of patient characteristics, administered drugs, and study design (6% for cabozantinib to 27%–100% for lenvatinib).^5^

**Figure 5:**
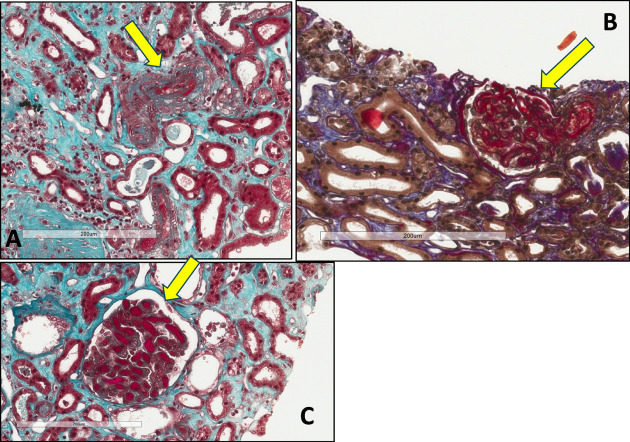
**Kidney biopsy findings in a patient on anti-VEGF therapy: TMA.** Histological examination of kidney biopsy showing TMA in a patient on anti-VEGF treatment. (A) The yellow arrow illustrates in high magnification (200 *μ*m) a thrombosed vessels with fibrinoid necrosis of the vessel wall in trichrome staining. (B) The yellow arrow illustrates in high magnification (200 *μ*m) a congested glomerulus in trichrome staining. (C) The yellow arrow illustrates in high magnification (200 pm) a preglomerular arteriole thrombotic lesion in AFOGH staining. AFOGH, acid–fuchsin orange G; TMA, thrombotic microangiopathy.

The pathogenesis of proteinuria is not fully understood. Podocytes express VEGF while VEGFR is present on the surface of both glomerular capillary endothelial cells and podocytes. This VEGF-mediated interaction promotes podocyte–endothelium crosstalk, enhancing the survival, proliferation, and/or differentiation of adjacent fenestrated glomerular capillary endothelial cells. Disruption of the podocyte–endothelial–VEGF axis results in loss of endothelial fenestrations, endotheliosis, podocytes loss, and subsequent proteinuria.^5,8^ Pathological findings often include kidney-limited thrombotic microangiopathy (TMA) (Figure 5) in patients treated with VEGF inhibitors and minimal change disease or focal segmental glomerulosclerosis with mTKIs.^5,6^

### Management

Early identification, prevention, and treatment of HTN are crucial for maintaining proper drug intensity and ensuring therapeutic success. Before initiating antiangiogenic agents, managing preexisting HTN is essential and patients should undergo frequent BP monitoring. First-line therapy for antiangiogenic-induced HTN includes angiotensin-converting enzyme inhibitor (ACEi), angiotensin II receptor blockers, *β*-blockers, and nondihydropyrimidine calcium channel blockers.^8^

Proteinuria often is mild and asymptomatic, not requiring drug discontinuation or dose changes. The first-line treatment of mild cases of proteinuria is ACEi. The development of class-defined adverse events during treatment may correlate with improved survival.^6,9^ Symptomatic proteinuria (edema, weight gain, and pleural effusion), nephrotic syndrome, TMA, or AKI may demand treatment interruption. In such cases, drugs can be reintroduced at reduced doses or noncontinuous schedules upon improvement in the Common Toxicity Criteria for Adverse Event (CTCAE) grade of proteinuria.^8^ Regular monitoring of kidney functions and proteinuria before initiating treatment and at each cycle is crucial.^10^

## FGFR Inhibitors

### Mechanism of Action/Kidney Toxicity

In tumors characterized by genetic alterations in the fibroblast growth factor (FGF) pathway, use of FGFR inhibitors has demonstrated therapeutic efficacy. FGFR is a transmembrane protein present ubiquitously on the cell surface in normal tissues throughout the body. When the ligand binds to the receptor, it triggers the activation of numerous oncogenic pathways that promote cellular processes, such as proliferation, maturation, survival, differentiation, migration, and angiogenesis.^11^ The FGF pathway, particularly involving the FGFR1 receptor, FGF23 ligand, and membrane Klotho in the proximal renal tubule, plays a role in phosphorus homeostasis. In normal circumstances, FGF binding its receptor inhibits sodium-phosphorus cotransporters, thus reducing kidney phosphorus reabsorption. Blocking FGFR disrupts these homeostatic mechanisms, leading to increased serum phosphorus levels.^11^ Hyperphosphatemia is, therefore, a common adverse event associated with FGFR inhibitors, observed in approximately 74% of patients.^12^ Such patients might have no symptoms to at most metastatic calcinosis.^13^ These patients exhibit abnormal serum phosphate levels and either normal or elevated levels of calcium; elevated phosphate levels may stimulate parathyroid hormone secretion, resulting in increased serum calcium levels and potential calcinosis cutis.^14^ Furthermore, some patients may develop calciphylaxis, characterized by tissue mineralization of small vessel walls due to disturbances in calcium and/or phosphate metabolism.^15^

### Management

Although calciphylaxis is rare, clinicians should evaluate patients' underlying risk factors and provide appropriate counseling.^13^ Further studies are needed to identify more effective strategies for phosphorus control to prevent interruptions in oncological drug therapy. The initial treatment recommendation is to reduce dietary phosphorus intake. When phosphorus levels exceed ≥7 mg/dl, it becomes essential to introduce phosphate-lowering therapies, such as binders and phosphaturic agents, to prevent potential interruptions in erdafitinib treatment. In the absence of confirming data in the literature, it is reasonable to suggest replacing warfarin anticoagulants in patients who develop hyperphosphatemia to prevent the development of calciphylaxis. Despite existing strategies, reducing the dose of erdafitinib is often necessary for effective management of phosphorus levels.^16^

## Tyrosine Kinase Inhibitors (cMET, EGFR, and Fms-Like Tyrosine Kinase 3 Anaplastic Lymphoma Kinase)

### Mechanism of Action/Kidney Toxicity

Tyrosine kinase inhibitors (TKIs) are used in the treatment of several types of cancers, including chronic myeloid leukemia (CML), non–small cell lung cancer, gastrointestinal stromal tumors, and RCC. Toxicity is dependent on the target of the TKI. In a phase 1 dose escalation study, cMET drugs were shown to cause obstructive nephropathy, crystal nephropathy, and formation of insoluble metabolites.^17^ Interruptions in the regulation of EGFR lead to several malignancies of the head/neck, breast, pancreas, and lung. Small molecule TKIs, such as erlotinib, gefitinib, brigatinib, icotinib, and afatinib, are known to cause a wide range of nephrotoxicity from IgA nephropathy to tumor lysis syndrome (TLS) (Table 2).

**Table 2:** Kidney toxicities, treatment options, and dose changes for molecular targeted therapies

Target	Agent	Pharmacokinetics^18^ t _1/2_/Renal Metabolism/Clearance	Kidney Toxicity	Treatment	Dose Change	References
BCL-2 inhibitor	Venetoclax	19–26 h/none/hepatic	TLS AKI	Hydration Allopurinol	Slow escalation of dose	See details in text
Epigenetic inhibitors	Belinostat Panobinostat Vorinostat Romidepsin Decitabine Azacitidine Ivosidenib Enasidenib	1.1 h/40%/hepatic 30 h/2%/unk 2 h/<1%/unk 3 h/unk/8.4 L/h 0.62 h/<1%/125 ml/h per m^2^ 41 min/<1%/167 L/h 58 h/17%/5.6 L/h 7.9 d/11%/0.70 L/h	AKI Hemorrhagic cystitis azotemia Collapsing glomerulopathy TMA Renal tubular acidosis Salt wasting Differentiation syndrome Pseudo-AKI	Cessation of therapy Supportive care High dose steroids for collapsing GN Fluid/electrolyte replacement	Dose resumed after toxicity resolves	See details in text
PIs	Bortezomib Carfilzomib Ixazomib	40–193 h/unk/102–112 L/h <1 h/unk/151–263 L/h 9.5 d/62%/unk	TMA, AIN TMA, prerenal, TLS, ATN None reported	? plasmapheresis ? eculizumab in reports: Insufficient data	Reduce dose by 25% if GFR <20 ml/min	19
PARP inhibitor	Olaparib Niraparib Talazoparib	6.10 h/44%/4.55 L/h 36 h/47%/16.2 L/h 90 h/68.7%/6.45 L/h	Increases in creatinine Pseudo-AKI	Check cystatin-C to confirm pseudo-AKI	Dose reduction to 200 mg BID for CrCl 31–50 ml/min	20
Biological agent	IFN	Unk/unk/9.4–28.9 ml/min	Proteinuria Minimal change disease TMA ATN	Discontinue	None	21,22
Biological agent	IL-2	unk	Capillary leak syndrome AKI Hypovolemia/prerenal	Cessation of therapy Supportive treatment	Avoid in older/CKD/concomitant nephrotoxins/risk of prerenal azotemia	23,24
XPO 1 inhibitor	Selinexor	6–8 h/unk/17.9 L/h	Hyponatremia prerenal, Hypovolemia SIAD	IV fluids d/c antiemetics/antidepressants/diuretics Salt tablets	Discontinuation Medication	25
Bruton TKI	Ibrutinib	4–6 h/7.8%/112–159 ml/min	AKI TLS	Hydration Monitor kidney functions	None	26
CD22-directed cytotoxin	Moxetumomab	1.4 h/approximately 100%/25 L/h	AKI Proteinuria	Monitor kidney functions	Delay new cycle if CTCAE grade 3 AKI	27
CD30-directed antibody	Brentuximab	4–6 d/unk/unk	Worsens already present kidney impairment	Stop drug and go for HSCT if CR	Avoid if CrCl <30 ml/min	28
ALK/cMET	Imatinib Sunitinib Ceritinib Crizotinib Alectinib Brigatinib Capmatinib	18–40 h/13%/8–14 L/h 40–110 h/16%/34–62 L/h 41 h/1.3%/33–88 L/h 42 h/2.3%/60–100 L/h 33 h/0.5%/82 L/h 25 h/86%/12.7 L/h 6.5 h/22%/24 L/h	Acquired Fanconi syndrome Hypophosphatemia ATN Interstitial mononuclear cell infiltration including eosinophils TMA Kidney cysts (new and progression of preexisting cysts) hematuria Prerenal Pseudo-AKI	Electrolyte repletion Regular imaging of kidney cysts Drainage of cysts when required Check cystatin-C to confirm pseudo-AKI^29^	Discontinuation of treatment If CrCl <30, reduce dose of crizotinib to 250 daily from BID Electrolyte repletion Regular imaging of kidney cysts Drainage of cysts when required	30–36
EGFR	Afatinib Erlotinib Gefitinib Brigatinib Icotinib Osimertinib	37 h/4.3%/1530 ml/min 36.2 h/8%/unk 48 h/<4%/595 ml/min 25 h/25%/12.7 L/h 5.5 h/9%/13.3±4.78 L/h 48 h/14%/14.3 L/h	Minimal change disease IgA nephropathy Crescent formation Tubular injury Immune-complex GN ATN TLS	Holding drug Immunosuppression	No dose changes needed	37,38
BCR-ABL	Imatinib Dasitinib	18–40 h/13%/14 L/h 3–5 h/4%/363.8 L/h	ATN TLS Proximal tubulopathy Fanconi syndrome Hypophosphatemia Proteinuria	Discontinuation Switching to alternate TKI	No dose adjustment	39–43
BTK	Ibrutinib	4–6 h/7.8%/112–159 ml/min	Interstitial nephritis	Early detection Glucocorticoids	Discontinuation	44
BRAF/MEK/CDK4/6	Vemurafenib Dabrafenib Ribociclib Palbociclib Abemaciclib	57 h/1%/31 L/d 10 h/23%/34.4 L/h 32.6 h/unk/unk 29 h/17.5%/63.1 L/h 18.3 h/3%/26 L/h	ATN Interstitial fibrosis Interstitial nephritis Peripheral eosinophilia Proteinuria Hyponatremia Hypokalemia Pseudo AKI	Discontinuation of drug	Discontinuation of drug until grade 0/1, then 25% reduced dose No changes with pretreatment CrCl <30 ml/min except for ribociclib (reduce to 200 mg daily)	45–47
mTOR	Everolimus Temsirolimus	30 h/5%/unk 17.3 h/4.6%/16.2 L/h	ATN	Monitoring of proteinuria Addition of ACE/ARB Discontinuation of drug	Holding treatment until resolution Discontinuation permanently with CTCAE grade 4 proteinuria or AKI	48,49

ACE, angiotensin-converting enzyme; AIN, acute interstitial nephritis; ALK, anaplastic lymphoma kinase; ARB, angiotensin II receptor blocker; ATN, acute tubular necrosis; BCL-2, B-cell lymphoma 2; BCR-ABL, gene sequence; BID, twice daily; BRAF, v-Rapidly accelerated fibrosarcoma viral oncogene homolog B1; BTK, Bruton’s tyrosine kinase; CDK, cyclin-dependent kinase; cMET, c-mesenchymal–epithelial transition; CR, complete remission; CrCl, creatinine clearance; CTCAE, Common Toxicity Criteria for Adverse Event; EGFR, epidermal growth factor receptor; HSCT, hematopoietic stem cell transplantation; IFN, interferon; IL-2, interleukin-2; MEK, mitogen-activated protein kinase; mTOR, mechanistic target of rapamycin; PARP, poly-adenosine diphosphate ribose polymerase; PI, proteosome inhibitor; SIAD, syndrome of inappropriate antidiuresis; TKI, tyrosine kinase inhibitor; TLS, tumor lysis syndrome; TMA, thrombotic microangiopathy; unk, unknown; XPO inhibitor, exportin-1 inhibitor.

Gilteritinib, an Fms-like tyrosine kinase 3 inhibitor, used in the treatment of refractory/relapsed acute myeloid leukemia can cause AKI (14%) through prerenal injury due to diarrhea.^50^ Other clinical features include cytopenia and transaminitis. One case was reported of CTCAE grade 4 toxicity requiring temporary hemodialysis.^50^

Advanced non–small cell lung cancer treated with crizotinib and ceritinib cause complex kidney cysts (in 4%), prerenal injury, and electrolyte abnormalities (Table 2). With ceritinib, hypophosphatemia was seen with 10% of patients, hypomagnesemia in 8%, and elevation in creatinine in 11%. Pseudo-AKI has been reported with some TKIs, such as anaplastic lymphoma kinase inhibitors and mesenchymal–epithelial transition inhibitors^30,31^ (Table 2).

The renal transporters that constitute the tubular creatinine secretion pathway are OCT2/OCT3/OATP2 at the basolateral and MATE-1/MATE-2k at the luminal surface of tubular epithelial cells. Other than creatinine, these renal transporters have other substrates that are pharmacologic agents, such as anaplastic lymphoma kinase inhibitors. This interference with the creatinine tubular secretion pathway can cause false rise in serum creatinine (SCr) level and reduction in eGFR, which can be interpreted falsely as AKI (pseudo-AKI).^29^ Alternative filtration markers, such as cystatin-C, can be used to noninvasively unmask a pseudo-AKI associated with these agents. Ideal filtration markers that exhibit very accurate measurements of GFR and can differentiate between true AKI and pseudo-AKI would be the use of exogenous filtration markers, such as radionuclides (^51^Cr-EDTA, ^99m^Tc-DTPA) and contrast agents (iothalamate, iohexol). These modalities are not readily available in many centers.^29^

### Management

AKI was seen in patients receiving higher, daily doses of cMET agents. Treatment required dose reduction, temporarily holding the drug, or immunosuppression.^51^ With crizotinib, because 22% is excreted in urine, the dose should be reduced to 250 mg daily from twice daily in patients with creatinine clearance <30 ml/min with rapid kidney recovery seen after discontinuation.^52^ For events associated with ceritinib, recommendations include regular monitoring of electrolytes and kidney function.^48^ While some kidney cysts required percutaneous drainage, close monitoring is recommended for most.^48^

## Nonreceptor TKIs (Gene Sequence, Bruton’s TKI, Janus Kinase)

### Mechanism of Action/Kidney Toxicity

Kidney toxicities of gene sequence TKIs seem to be drug specific. Imatinib is currently used for gastrointestinal stromal tumors and CML.^48^ Degree of change in eGFR seems to be related to the duration of treatment.^39^ Imatinib can cause TLS, Fanconi syndrome, and proximal tubular dysfunction and toxic tubular damage by inhibiting platelet-derived growth factor receptor, which plays a role in tubular regeneration after acute tubular necrosis (ATN).^39^ In a study conducted in 100 patients with CML treated with imatinib, 7% experienced AKI and 12% developed CKD.^53^ Hypophosphatemia was seen in 10% patients in one case series because of low vitamin D 25-hydroxy and hypocalcemia.^54,55^ Imatinib-resistant CML can be treated with dasatinib, a second-generation TKI with only three published case reports of AKI.^56–58^ After 11 months of therapy, some patients with acute myeloid leukemia treated with 400 mg/d of imatinib and patients with CML treated with dasatinib at a dose of 100 mg/d experienced SIAD-induced hyponatremia.^59^ Ibrutinib is a Bruton’s tyrosine kinase inhibitor used mainly in the treatment of chronic lymphocytic leukemia (CLL) and Mantle cell lymphoma can cause nonoliguric AKI and proteinuria through acute interstitial nephritis (AIN).^44,60^

Ruxolitinib, tofacitinib, and baricitinib comprise the Janus kinase inhibitors used in the treatment of chronic hematopoietic neoplasms. Although liver toxicities have been reported, no kidney toxicities have been reported.^61^

### Management

Current strategies include discontinuation, reducing dose, or switching to an alternate TKI.^62^ Early detection is crucial and includes discontinuation of the drug and administration of glucocorticoids when warranted. No dose adjustments have been required for Janus kinase inhibitors.

## Serine/Threonine Kinase Inhibitor (v-Rapidly Accelerated Fibrosarcoma Viral Oncogene Homolog B1/Mitogen-Activated Protein Kinase, Cyclin-Dependent Kinase 4/6, and Mechanistic Target of Rapamycin)

### Mechanism of Action/Kidney Toxicity

Malignancies that are caused by v-Rapidly accelerated fibrosarcoma viral oncogene homolog B1 (BRAF) mutations, such as metastatic melanoma, can potentially be treated with BRAF inhibitors, such as vemurafenib and dabrafenib. Vemurafenib can cause AKI, proteinuria, rash, and peripheral hypereosinophilia. Vemurafenib decreased GFR by 20%–74% in a case series of eight patients. Patients with risk factors of developing kidney toxicities are those with the use of nonsteroidal anti-inflammatory agents, HTN, diabetes mellitus, CKD, recent use of contrast agents, or recent use of nephrotoxic standard chemotherapy.^45^ The fact that V600 mutation that BRAF inhibitors treat is more commonly noted in men could explain male predominance.^45^ In a 1-year data from FAERS, 13 cases of AKI were reported with dabrafenib.^63^ In general, this drug has been shown to be less kidney toxic. Interstitial nephritis manifests during the first couple of weeks, and tubular toxicity has been seen with longer treatment durations.^45^

Ribociclib belongs to the cyclin-dependent kinase 4/6 family and can cause AKI in nearly 60% of patients as per FAERS data.^46^ Of the six patients biopsied in a study by Gupta *et al.*, most had ATN and one had AIN.^46^ Early trials of palbociclib and ribociclib did not describe the incidence of AKI, whereas clinical trials of abemaciclib have reported that up to 25% of patients experienced a potentially reversible rise in SCr without actually changing GFR.^46^ At therapeutic concentrations, abemaciclib and its major circulating metabolites would inhibit OCT2 and MATE1/2-K clinically, resulting in elevations in SCr are a result of inhibition of active tubular secretion of creatinine and not a reduction in kidney function (pseudo-AKI).^64^

Everolimus and temsirolimus are two mechanistic target of rapamycin agents that are used in the treatment of RCC, refractory hormone receptor–positive breast cancer, and lymphomas. Kidney toxicities include proteinuria and AKI in patients with CKD through ATN, disruption of the VEGF pathway, inhibition of HIF-alpha, decreased uptake of albumin in proximal tubules, and increased glomerular macrophages. Fifty-seven percent patients had worsening creatinine levels with temsirolimus.^65^ Patients with RCC who had impaired kidney functions were at higher risk of AKI. Both drugs can cause different degrees of hyponatremia through aldosterone resistance.^66,67^ Temsirolimus can also cause phosphate wasting through ATN. ^68^

### Management

Although kidney function improves in some patients despite continuation of therapy with BRAF inhibitors, in general, the drug should be discontinued. Patients have recovery of kidney function with discontinuation of ribociclib.^69^ For grade 3 CTCAE toxicity, treatment methods include holding the drug, discontinuation of the drug with CTCAE grade 4 proteinuria/AKI, and addition of ACEi/angiotensin II receptor blocker for proteinuria.^48^

## B-Cell Lymphoma 2 Inhibitors (Venetoclax)

### Mechanism of Action/Kidney Toxicity

Venetoclax, an oral highly selective inhibitor of the B-cell lymphoma 2 family, was approved for the treatment of CLL and acute myeloid leukemia and off-label use for relapsed/refractory Mantle cell lymphoma. Venetoclax has been associated with a high incidence of TLS, causing AKI and severe electrolyte abnormalities. The incidence was found to be 10%–18% in early phase 1 studies.^70^ Risk is higher if either started at a very high dose or the dose is escalated too quickly in patients with moderate/large tumor burden or relapsed/refractory CLL.

### Management

TLS prophylaxis is performed by slow dose escalation starting at 20 mg daily and gradually increasing to finally a dose of 400 mg over 5 weeks.^71^ Avoiding concurrent start of anti-CD20 mAbs is required. If needed, obinutuzumab is started before, and rituximab is started after venetoclax. Regular monitoring of TLS laboratory test results is needed, and those at risk should be started on hypouricemic medications and hydration 2–3 days before the start of therapy. Patients relapsing after B-cell receptor pathway inhibitor treatment frequently have proliferative disease, requiring a faster time to target dose than this scheme allows, and need rapid dose escalation (RDE). Analysis of 33 patients with CLL who underwent venetoclax RDE after prior B-cell receptor pathway inhibitor treatment showed that 52% developed laboratory TLS and 15% developed clinical TLS, all because of kidney injury. TLS was seen in more patients with a higher initial tumor burden.

## *Epigenetic Inhibitors* (Azacitidine, Decitabine, Belinostat, Panobinostat, Vorinostat, Romidepsin, Ivosidenib, and Enasidenib)

### Mechanism of Action/Kidney Toxicity

Epigenetic inhibitors, such as DNA methyltransferase inhibitors and histone deacetylase inhibitors (HDACis), have been well established in treating hematological malignancies. They are known to induce cell and compound-specific nephrotoxicity.^72^ 5-Azacytidine induces kidney tubular acidosis in patients with advanced acute leukemia.^73^ HDACis induce apoptosis in renal tubular cells.^74^ DNA methyltransferase inhibitors and HDACis cause increased nephrotoxicity by inducing increased oxidative stress. These studies have determined the cytotoxicity of the epigenetic inhibitors alone and in combination with various chemotherapeutics on prostate cancer and kidney cells.^72^ Decitabine-induced TMA has been reported.^75^ Nephrotoxicity of decitabine is mild, rare, and dose dependent. In phase 2 clinical trials, decitabine can lead to direct injury to the endothelium.^75^ Clinical trials with decitabine-associated AKI revealed one patient recovered kidney functions within a week of drug withdrawal. Another patient had worsening kidney functions with a second course, late recovery with withdrawal, and finally biopsy-proven AIN.^76^ In a study of 22 patients receiving 33 courses of 5-azacytidine, during 29 courses (88%) of treatment, polyuria, glucosuria, and changes in bicarbonate or phosphorus levels were detected. Polyuria, salt wasting, and orthostatic hypotension occurred in 21% of treatment courses. These results suggested proximal and distal tubular damage.^73^ Possible inhibition of creatinine transporters in proximal tubular cells by azacytidine without affecting kidney functions has been reported as well.^77^

A novel class of epigenetic drugs, so-called mutant isocitrate dehydrogenase-2 inhibitor (mIDHi), has been developed. Two mIDHis, ivosidenib and enasidenib, have been approved, and several other mIDHis are being tested in preclinical and clinical trials. Enasidenib was approved for use in refractory acute myeloid leukemia. Data from multicenter, open-label, phase 1/2 study identified 11.7% as having isocitrate dehydrogenase (IDH)-differentiation syndrome (IDH-DS). Of the 33 patients, 14 had AKI. The onset of symptoms ranged from 7 days to 5 months.^78^ In another study of IDH-DS, induced AKI was seen in 17%–24% of patients.^79^

### Management

Drug therapy cessation is required in those with AKI. In those with tubular dysfunction, intensive fluid and electrolyte replacements were part of treatment. Romidepsin-induced collapsing glomerulonephritis was treated with high-dose steroids after cessation of drug therapy. In patients with IDH-DS, enasidenib was stopped; they were treated with dexamethasone and hydroxyurea for co-occurring leukocytosis and treated for TLS.

## *Proteosome Inhibitors* (Bortezomib, Carfilzomib, and Ixazomib)

### Mechanism of Action/Kidney Toxicity

**Figure 6:**
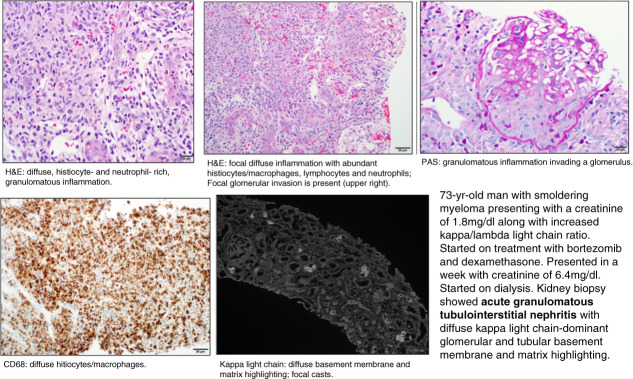
**Kidney biopsy findings in a patient on PI: acute granulomatous tubule-interstitial nephritis.** H&E, hematoxylin and eosin; PAS, periodic acid–Schiff; PI, proteosome inhibitor.

Proteosomes inhibit the ubiquitin–proteosome system regulating the growth of normal and tumor cells. Malignant cells are more sensitive to proteosome inhibitors (PIs) than normal cells.^80^ Bortezomib has been known to cause TMA in several case reports and AIN with granuloma formation (*e.g*., Figure 6).^80^ The decrease in NF-κB levels in the nucleus leads to decreased VEGF production potentially predisposing to TMA.^81^ In a study of 276 patients with multiple myeloma (MM), development of AKI after treatment with bortezomib was observed in the absence of TLS.^82^ Multivariant analysis revealed that lower baseline GFR, lower albumin levels, concomitant amyloidosis, and use of acyclovir during bortezomib treatment were independently associated with higher risk of AKI. Carfilzomib, which was approved for refractory MM, was found to cause AKI in 25% of patients in a phase 2 study. There have been reports of TMA, TLS, and ATN as well. The onset of TMA could range from 2 days to 17 months. It has been proposed that the early-onset TMA is an immune-mediated and the late onset a dose-dependent toxic mechanism and that plasmapheresis is unlikely to show benefit. Ixazomib has been approved for newly diagnosed and refractory MM. No known kidney toxicities have been reported.^83^ Information from the FOCUS trial reported several more cases of HTN and TMA reported with carfilzomib than bortezomib.^80^

### Management

Bortezomib-induced AIN is treated by discontinuation of the offending agent, initiation of steroids, and rarely requiring cyclophosphamide, as reported by Cheungpasitporn *et al.*^84^ TMA is treated with discontinuation of the offending drug and supportive care. Plasmapheresis has not shown to have any clinical benefit.^81^ Of the 11 patients described by Yui *et al.*, four patients were treated with plasma exchange, and three patients received eculizumab. Three patients died within 30 days of TMA diagnosis. Nine patients had resolution of TMA without evidence of hemolysis after withdrawal of PI. Two patients had stabilization of laboratory values but persistent evidence of hemolysis despite medication withdrawal. One patient had recurrence of TMA with rechallenge of PI.^83^

## *Poly-Adenosine Diphosphate Ribose Polymerase Inhibitors* (Olaparib)

Inhibitors of poly-adenosine diphosphate ribose polymerase are approved for the treatment of breast cancer gene (BRCA)-mutated breast cancer and platinum-sensitive relapsed epithelial ovarian cancer and for maintenance therapy for pancreatic cancer with BRCA-1 or BRCA-2 mutation. See Table 2 for toxicities and treatment.^20^

## Other Agents

Brentuximab, ibrutinib, moxetumomab, selinexor, IFNs, and IL 2 are summarized in Table 2.^21–28^

## Conclusion

New anticancer medications are rapidly evolving, offering patients with previously resistant cancers the promise of more effective therapies capable of extending their lives. However, adverse kidney consequences develop in treated patients with underlying risk factors, requiring the nephrology community to be familiar with their nephrotoxic effects. Through multidisciplinary and collaborative efforts between oncology and nephrology, the promise of cancer treatments can be realized while minimizing kidney‐related complications that have both short‐term and long‐term consequences.
